# A machine‐learning algorithm to grade heart murmurs and stage preclinical myxomatous mitral valve disease in dogs

**DOI:** 10.1111/jvim.17224

**Published:** 2024-10-21

**Authors:** Andrew McDonald, Jose Novo Matos, Joel Silva, Catheryn Partington, Eve J. Y. Lo, Virginia Luis Fuentes, Lara Barron, Penny Watson, Anurag Agarwal

**Affiliations:** ^1^ Department of Engineering University of Cambridge Cambridge United Kingdom; ^2^ Department of Veterinary Medicine University of Cambridge Cambridge United Kingdom; ^3^ North Downs Specialist Referrals Bletchingley United Kingdom; ^4^ Royal Veterinary College Hertfordshire United Kingdom; ^5^ Davies Veterinary Specialists Hitchin United Kingdom

**Keywords:** auscultation, cardiology, dog, electronic stethoscope, stage B

## Abstract

**Background:**

The presence and intensity of heart murmurs are sensitive indicators of several cardiac diseases in dogs, particularly myxomatous mitral valve disease (MMVD), but accurate interpretation requires substantial clinical expertise.

**Objectives:**

Assess if a machine‐learning algorithm can be trained to accurately detect and grade heart murmurs in dogs and detect cardiac disease in electronic stethoscope recordings.

**Animals:**

Dogs (n = 756) with and without cardiac disease attending referral centers in the United Kingdom.

**Methods:**

All dogs received full physical and echocardiographic examinations by a cardiologist to grade any murmurs and identify cardiac disease. A recurrent neural network algorithm, originally trained for heart murmur detection in humans, was fine‐tuned on a subset of the dog data to predict the cardiologist's murmur grade from the audio recordings.

**Results:**

The algorithm detected murmurs of any grade with a sensitivity of 87.9% (95% confidence interval [CI], 83.8%‐92.1%) and a specificity of 81.7% (95% CI, 72.8%‐89.0%). The predicted grade exactly matched the cardiologist's grade in 57.0% of recordings (95% CI, 52.8%‐61.0%). The algorithm's prediction of loud or thrilling murmurs effectively differentiated between stage B1 and B2 preclinical MMVD (area under the curve [AUC], 0.861; 95% CI, 0.791‐0.922), with a sensitivity of 81.4% (95% CI, 68.3%‐93.3%) and a specificity of 73.9% (95% CI, 61.5%‐84.9%).

**Conclusion and Clinical Importance:**

A machine‐learning algorithm trained on humans can be successfully adapted to grade heart murmurs in dogs caused by common cardiac diseases, and assist in differentiating preclinical MMVD. The model is a promising tool to enable accurate, low‐cost screening in primary care.

**Abbreviations**
AS: aortic stenosis
AUC: area under the receiver operating characteristic curve
CI: confidence interval
DCM: dilated cardiomyopathy
LA/Ao: left atrial to aortic root ratio
LVIDD: left ventricular internal diameter in diastole
LVIDDN: body weight‐normalized left ventricular internal diameter in diastole
MMVD: myxomatous mitral valve disease
NT‐proBNP: N‐terminal pro‐B‐type natriuretic peptide
PDA: patent ductus arteriosus
PS: pulmonic stenosis
ROC: receiver operating characteristic

## 1 INTRODUCTION

Auscultation is a key component of an initial physical examination of a dog.^1^ The identification of a heart murmur is a sensitive indicator of many heart diseases, with murmur intensity shown to correlate with the severity of myxomatous mitral valve disease (MMVD),^2^, ^3^, ^4^ pulmonic stenosis, and subaortic stenosis.^5^ Myxomatous mitral valve disease is the most common cardiac disease in adult dogs,^2^, ^6^, ^7^ where the presence of a murmur without signs of heart failure indicates the dog is in stage B.^6^ In stage B dogs where there also is evidence of heart enlargement (stage B2), administering pimobendan is recommended to delay the onset of heart failure.^6^, ^7^ Differentiating stages accurately currently requires echocardiography. However, recent studies have attempted to discriminate stage B1 and B2 MMVD using multivariable models that combine biomarkers and physical examination findings, repeatedly demonstrating that murmur intensity, assessed by a cardiologist, is an important predictive variable.^2^, ^3^, ^8^

An accurate assessment of a murmur's intensity could provide a valuable indication of whether time‐consuming and expensive diagnostic tests, such as radiography or echocardiography, are required. This assessment is particularly important in general practice settings with limited resources or where owners face financial constraints. However, murmur grading can be subjective and has significant inter‐observer variability,^9^, ^10^ which limits its use in diagnostic criteria. Accurate auscultation with a stethoscope requires substantial skill and confidence that has declined in recent years.^1^

Electronic stethoscopes are increasingly available in veterinary practice and can record heart sounds for digital analysis.^11^, ^12^, ^13^ This practice opens the possibility of using signal processing and machine‐learning algorithms to automatically detect and grade heart murmurs, potentially minimizing inter‐observer variability and lowering the skill barrier of auscultation. In humans, considerable work has been done on designing machine‐learning algorithms to analyze heart sounds, partially driven by large open‐access datasets.^14^, ^15^, ^16^, ^17^ However, similar datasets are not available for dog heart sounds, and so developments have been limited. Heart sounds are not routinely collected and digitally stored, unlike other cardiac tests such as echocardiography or electrocardiography, which means customized studies are required. Two previous studies have aimed to discriminate innocent and pathological murmurs in datasets of 27^18^ and 77^19^ dogs using hand‐crafted frequency analysis. The size of these earlier datasets limits the scope of analyses and the statistical significance of results.

We aimed to design and evaluate a machine‐learning algorithm to grade heart murmurs in electronic stethoscope recordings and stage preclinical MMVD. Our objectives were to (a) collect a new large dataset of heart sound recordings along with the clinical diagnoses from a diverse population of dogs, (b) train a machine‐learning algorithm to detect and grade dog murmurs, transferring algorithms trained on larger datasets from humans, and (c) assess the accuracy of gradings compared to expert cardiologists and determine if the predictions provide useful distinction between stage B1 and B2 MMVD.

## 2 MATERIALS AND METHODS

This study adheres to the STARD 2015 guidelines for reporting diagnostic accuracy studies.^20^ A table summarizing this adherence on a point‐by‐point basis is available in Supporting Information.

### 2.1 Recruitment

Dogs undergoing routine echocardiography at 4 veterinary referral centers in the United Kingdom (Queen's Veterinary School Hospital, University of Cambridge; Queen Mother Hospital for Animals, Royal Veterinary College; North Downs Specialist Referrals; Davies Veterinary Specialists) between 2019 and 2023 were recruited. The study was approved by the Ethics and Welfare Committee of the Department of Veterinary Medicine, University of Cambridge (CR582, CR372). The local ethics committee of each recruitment center approved the study. All owners consented to the collection and storage of their dog's anonymized data, which was stored using REDCap.^21^, ^22^

### 2.2 Clinical evaluation

Every dog was examined by a board‐certified veterinary cardiologist or a supervised cardiology resident. A commercially available Conformité Européene (CE) marked electronic stethoscope (3M Littmann 3200 or Eko DUO) was used to make 15‐second recordings at the left apex, left base, and right‐sided auscultation positions. The cardiologist or resident graded the Levine intensity (I‐VI) of any murmurs present at each auscultation site and noted the point of maximal intensity of the murmur. The Levine grades were later converted into a reduced 4‐level scale.^23^ The reduced scale, shown in Table 1, maintains clinical relevance while decreasing the number of outputs in the machine‐learning model and hence simplifying the analysis. The resting heart rate was recorded, and the heart rhythm was classified using electrocardiography. Body condition score was graded on a standard 9‐point scale.^24^ Existing clinical signs such as cough or exercise intolerance also were noted.

**TABLE 1:** Conversion between the 6‐level Levine murmur intensity scale and reduced murmur intensity scale, as previously proposed.^23^

Reduced grade	Levine grade	Definition
Soft	I/II	Murmur is softer than the S1/S2 heart sounds.
Moderate	III	Murmur is equal intensity to the S1/S2 sounds.
Loud	IV	Murmur is louder than the S1/S2 sounds.
Thrilling	V/VI	Murmur has a palpable thrill, regardless of intensity.

Echocardiography was performed on all dogs using standard views.^25^ Myxomatous mitral valve disease was defined as characteristic valvular thickening with mitral valve regurgitation on color Doppler.^7^ The left atrial to aortic root ratio (LA/Ao) and left ventricular internal diameter in diastole (LVIDD) were recorded, and the body weight‐normalized left ventricular internal diameter (LVIDDN) measurement was calculated as LVIDD (cm)/weight (kg)^0.294^.^26^ The MMVD cases were classified into B1, B2, C, and D stages using the American College of Veterinary Internal Medicine (ACVIM) consensus guidelines.^6^ As in previous studies,^2^, ^3^ the echocardiographic criteria alone were used to differentiate stage B1 and B2, with dogs with LA/Ao ≥1.6 and LVIDDN ≥1.7 classified as B2.^6^ Radiographs were not collected as part of the study and hence vertebral heart sums were not calculated. For the sub‐analysis of detecting MMVD, dogs with a normal heart and body weight <15 kg were classified as MMVD stage A. Dilated cardiomyopathy was defined as left ventricular systolic dysfunction and volume overload in the absence of any congenital or acquired heart disease or systemic disease capable of inducing similar changes.^27^ Pulmonic and aortic stenosis were defined, respectively, as increased transpulmonic and transaortic flow velocities on continuous wave Doppler. Patent ductus arteriosus was defined as a characteristic left‐to‐right shunt across a patent ductus arteriosus. Mitral valve dysplasia was defined as an abnormal mitral valve apparatus (eg, elongated and thickened mitral valve leaflets with short chordae tendineae) associated with valve regurgitation on color Doppler.

### 2.3 Allocation of study sample

**FIGURE 1:**
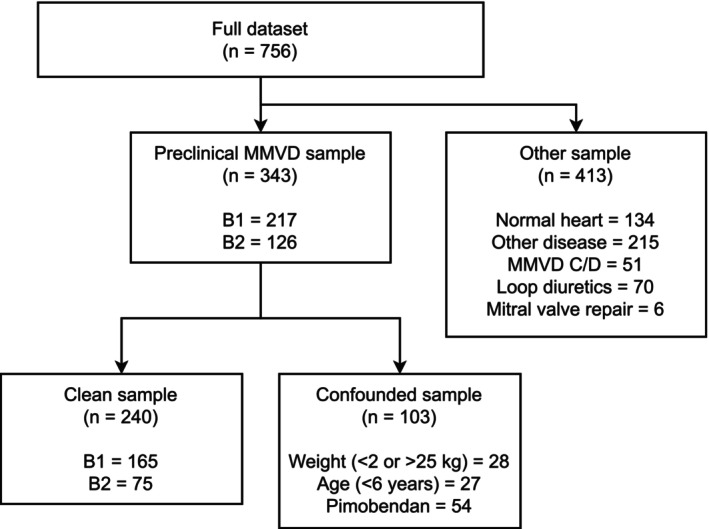
Flowchart showing separation of data for the different analyses in this work. Murmur detection and grading was evaluated using the full dataset (n = 756), having examples of normal hearts, MMVD, and other diseases. Differentiation of preclinical MMVD (stage B1 or B2) was evaluated using the clean and confounded sections of the MMVD sample (n = 343).

A total of 756 dogs was recruited. The full dataset was used to train and evaluate algorithms to grade and detect heart murmurs. To assess the performance of the algorithm at classifying preclinical MMVD, a reduced dataset was created following a previously described protocol.^2^ Dogs with a normal heart, other cardiac diseases, congestive heart failure (stage C/D MMVD), or on loop diuretics (furosemide or torasemide) were excluded from this subanalysis. This resulted in a preclinical MMVD sample of 343 cases. Following the previously described method and nomenclature,^2^ this dataset was further partitioned into a clean sample (n = 240) and a confounded sample (n = 103). The confounded sample included patients with body weight outside of the 2‐25 kg range, < 6 years of age, or dogs that were medicated with pimobendan. These variables previously were identified as potential confounders.^2^ This partitioning of the data is shown in Figure 1.

### 2.4 Machine learning analysis

All analyses were performed with Python using the open‐source PyTorch, SciPy and Scikit‐Learn libraries.

To aid in model training, a transfer learning approach was used,^28^ where a model trained to detect heart murmurs in humans was used as a starting point. The model, developed in a previous work,^29^ was trained on the open‐access challenge datasets from the PhysioNet 2016^17^ and PhysioNet 2022^14^ challenges. The model consists of 2 key stages. The first is feature extraction, where the heart sound recording is transformed into a normalized log‐spectrogram time‐frequency representation. This representation enables visual separation of the low‐frequency S1 and S2 heart sounds from higher‐frequency murmurs that may appear in systole or diastole. The second stage is a bidirectional recurrent neural network with gated recurrent unit^30^ cells trained to predict the presence of a murmur using the label provided by a human expert.

Hyperparameters of the feature extraction (eg, spectrogram parameters such as window length) and the neural network (eg, layer size, memory cell) were optimized solely based on the heart sound data from humans through 5‐fold cross‐validation. The model was trained with a cross‐entropy loss using the Adam^31^ optimizer. The final model weights were saved and then used as a starting point for the dog model.

The trained model then was extended and fine‐tuned to grade dog heart murmurs. The Levine grade of a heart murmur is an ordinal variable, in contrast to the binary murmur detection problem for which the human model was trained originally. The model therefore was adjusted to use multiple output nodes, with the final weight and bias of the human neural network cleared before fine‐tuning it on the dog data.

#### 2.4.1 Training and test data

The dog dataset (n = 756) was split into 2 equal training and test portions using a minimized stratification algorithm that balanced recruitment site, disease type (MMVD, normal, other), heart rate, maximum murmur intensity, body condition score, and body weight of patients. This 50% test split is higher than traditionally used in machine learning literature to give larger and more robust cohorts in subanalyses of congenital heart diseases and MMVD stages.

The human‐trained models were trained on the dog training data (n = 378) using a 5‐fold cross‐validation. The starting point for all model parameters was the optimized human version, and early stopping was used to prevent model overfitting. The fine‐tuned dog models then were evaluated on the withheld test set.

**FIGURE 2:**
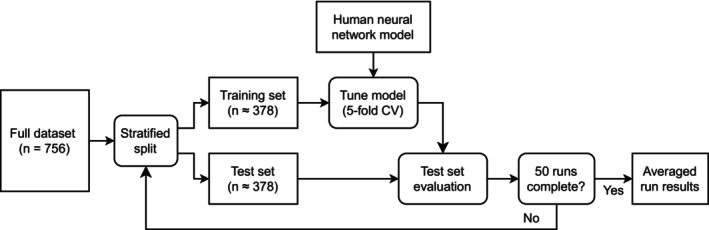
Nested evaluation procedure. The full patient dataset is split into training (50%) and test (50%) samples using a stratified minimization algorithm that balances key variables such as recruitment site, age, and disease. The human neural network model is then fine‐tuned on the dog training set and evaluated on the test set. The process was then repeated 50 times to give a test distribution that matched the full dataset without overfitting.

A limitation of smaller medical datasets is that the selection of the test set is an important source of randomness in the test results. To account for this variability and provide appropriate confidence intervals (CIs), a repeated stratification process was used.^32^ After the first training and evaluation process was completed, the full dataset from dogs was split into new training and test sets using a new random seed, and the full model training and evaluation procedure was repeated. This process was repeated 50 times, generating 50 trained versions of the machine‐learning model. Because an equal training and test split was used in every repeat, each patient appeared in the test set approximately 25 times. The nested structure ensured that in each repeat, the new model only predicted results for data it had not seen during training. Doing so ensured that the test results were not biased by any overfitting of the model to its training data. Additionally, because all model hyperparameters were fixed on the dataset from humans, there was no risk of the model structure being optimized for the dog test set. Figure 2 shows an illustration of this nested evaluation procedure.

### 2.5 Statistical analysis

The nested evaluation procedure gave a set of 50 results on different stratified test sets. Iterative bootstrap sampling (with 1000 iterations) was further applied to each run to give an improved estimate of the final test sample distributions without making assumptions of normality. The bootstrapped distributions were used to give mean values and 95% CIs for continuous test results. A significance level of 5% was used. The normality of continuous variables was assessed by inspection of their histograms and the Shapiro‐Wilk test. Non‐parametric continuous data is reported as median and interquartile range. Categorical variables are reported as proportion and frequency.

Analysis of binary classification tasks (murmur detection and MMVD B1 vs B2 classification) was performed using receiver operating characteristic (ROC) curves. Individual ROC curves were generated for each bootstrapped sample and averaged ROC curves were generated by vertical averaging.^33^ For the murmur detection task, the grade assigned by the attending cardiologist was used as the gold standard. For the MMVD B1 vs B2 task, the ACVIM consensus echocardiographic criteria were used as the gold standard. In both cases, individual operating points on the ROC were specified in terms of their diagnostic sensitivity and specificity. Sensitivity and specificity are defined as the proportion of gold standard positive and negative cases correctly identified by the algorithm, respectively.

Murmur grading is an ordinal regression task and was analyzed in terms of micro‐averaged accuracy (eg, the proportion of recordings where the predicted grade from the algorithm matched the prediction from the expert).

## 3 RESULTS

### 3.1 Dataset

Of the 756 dogs recruited, 407 received a primary diagnosis of MMVD (B1 = 225, B2 = 131, C = 44, D = 7), 215 of other diseases, and 134 of a normal heart. Sixty‐two dogs with a normal heart and body weight <15 kg were classed additionally as stage A (at‐risk) of MMVD. The most common other primary diseases recorded were pulmonic stenosis (n = 49), patent ductus arteriosus (n = 34), aortic stenosis (n = 32), dilated cardiomyopathy (n = 24), and mitral valve dysplasia (n = 13). The most common breed was Cavalier King Charles Spaniel (n = 88), followed by Chihuahua (n = 55) and Labrador (n = 45).

**FIGURE 3:**
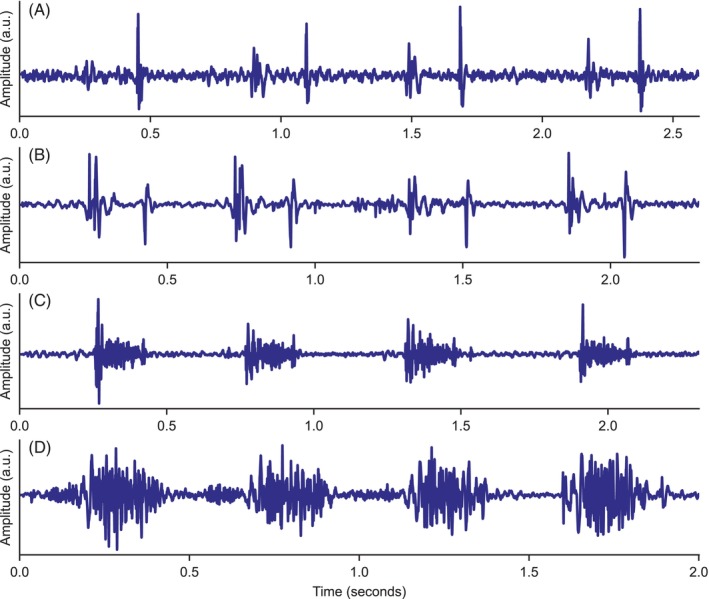
Example heart sound recordings, with 4 heartbeats shown for each case. (A) A left base recording of a normal heart with no murmur; (B) a left apical recording of a soft (I/VI) murmur in a dog with MMVD stage B1; (C) a left apical recording of a loud (IV/VI) murmur in a dog with MMVD stage B2; (D) a left base recording of a thrilling murmur from a dog with pulmonic stenosis. a.u., arbitrary units.

In all, 2258 recordings were made with a total audio duration of 618 minutes. All dogs were recorded at the left apex, but a small number lacked recordings at 1 (n = 8) or 2 (n = 1) of the other auscultation sites because of noise or other recording difficulties. Figure 3 shows some example recordings from dogs in the dataset with different murmur grades and diseases.

Table 2 shows the distribution of murmur grades and locations across the entire dataset, according to the cardiologist. The presence of a murmur of any grade was a sensitive indicator for cardiac disease: 98% of MMVD cases and every case of pulmonic and aortic stenosis had a murmur. Murmurs were present in 30% of normal hearts, but most (28%) were soft. In MMVD cases, 93% of murmurs had a point of maximal intensity over the left apex, as expected. In other conditions, such as pulmonic stenosis, aortic stenosis, and patent ductus arteriosus, the modal location was over the left base.

**TABLE 2:** Distribution of point of maximal intensity and grade of murmurs across the most common diagnoses in the dataset.

Variable	Normal	MMVD	PS	AS	PDA	DCM
Maximal intensity						
None	70% (94)	2% (7)	0% (0)	0% (0)	6% (2)	17% (4)
Soft (I/II)	28% (37)	21% (87)	6% (3)	28% (9)	24% (8)	46% (11)
Moderate (III)	2% (3)	28% (114)	22% (11)	28% (9)	6% (2)	29% (7)
Loud (IV)	0% (0)	34% (139)	20% (10)	34% (11)	9% (3)	8% (2)
Thrilling (V/VI)	0% (0)	16% (64)	51% (25)	9% (3)	56% (19)	0% (0)
Point of maximal intensity						
None	70% (94)	2% (7)	0% (0)	0% (0)	6% (2)	17% (4)
Left Base	16% (21)	1% (3)	84% (41)	72% (23)	68% (23)	12% (3)
Left Apex	13% (17)	93% (384)	16% (8)	25% (8)	24% (8)	71% (17)
Right Side	1% (2)	4% (15)	0% (0)	3% (1)	3% (1)	0% (0)

*Note*: Variables as reported as proportion (frequency). Maximum intensity is as recorded by the expert cardiologist over the 3 auscultation sites. The point of maximal intensity is shown only for cases with a murmur. Some murmurs are counted twice, as some cases had multiple diseases. Missing data: MMVD point of maximal intensity (n = 2).

Abbreviations: AS, aortic stenosis; DCM, dilated cardiomyopathy; PDA, patent ductus arteriosus; PS, pulmonic stenosis.

Table 3 further breaks down the key characteristics of dogs with a main diagnosis of MMVD. Seven percent of B1 and 38% of B2 cases were medicated with pimobendan at the time of recording. The distribution of age, body condition score, and sex was similar across B1 and B2 dogs. Left apex murmur intensity was again a useful discriminator between B1 and B2: 73% of B2 dogs had a loud or thrilling left apical murmur (equivalent to Levine grade IV or higher) compared with 23% of B1 cases.

**TABLE 3:** Breakdown of key characteristic of staged MMVD dogs.

Variable	A (n = 62)	B1 (n = 225)	B2 (n = 131)	C/D (n = 51)
Age (years)	4.4 (2.1, 8.2)	9.5 (7.8, 11)	10 (8.6, 12)	10 (9.2, 12)
BCS				
<5.0	71% (44)	57% (129)	54% (71)	76% (39)
5.0‐9.0	26% (16)	38% (85)	40% (52)	16% (8)
Breed				
CKCS	3% (2)	17% (38)	25% (33)	22% (11)
Sex				
Female entire	18% (11)	8% (18)	7% (9)	4% (2)
Female neut.	35% (22)	36% (80)	45% (59)	47% (24)
Male entire	24% (15)	21% (48)	12% (16)	12% (6)
Male neut.	23% (14)	35% (78)	34% (45)	35% (18)
Body weight (kg)	8.8 (5.3, 11)	9.8 (6.9, 15)	8.7 (6.0, 12)	9.3 (5.2, 13)
Pimobendan	0% (0)	7% (16)	38% (50)	73% (37)
Arrhythmia	23% (14)	26% (57)	29% (38)	43% (22)
LA/Ao	1.40 (1.30, 1.43)	1.42 (1.35, 1.50)	1.80 (1.70, 1.98)	2.29 (1.96, 2.69)
LVIDDN	1.52 (1.42, 1.59)	1.57 (1.44, 1.66)	1.88 (1.77, 1.99)	2.20 (1.95, 2.38)
Left apex murmur grade				
None	81% (50)	3% (7)	‐	‐
Soft (I/II)	19% (12)	39% (88)	2% (2)	2% (1)
Moderate (III)	‐	35% (79)	25% (33)	6% (3)
Loud (IV)	‐	21% (47)	49% (64)	49% (25)
Thrilling (V/VI)	‐	2% (4)	24% (32)	43% (22)

*Note*: Continuous variables are reported as median (interquartile range). Categorical variables are reported as proportion (frequency). Murmur grades are reported on the Levine scale (I‐VI) and grouped according to the simplified scheme of Rishniw.^23^ Missing data: stage A LA/Ao (n = 1), stage B1 age (n = 1), BCS (n = 25), sex (n = 4).

Abbreviations: BCS, body condition score; CKCS, Cavalier King Charles Spaniel.

### 3.2 Murmur grading

**FIGURE 4:**
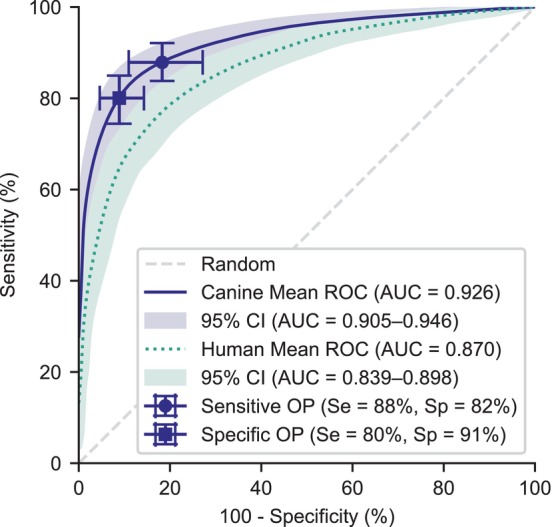
Receiver operating characteristic curve for binary murmur detection. The fine‐tuned canine model achieves a mean area under the curve (AUC) of 0.926 (95% CI: 0.905‐0.946). The original baseline human algorithm, before transfer learning, achieves an AUC of 0.870 (95% CI: 0.839‐0.898). Confidence intervals are generated through averaging individual runs and bootstrapping. Two example operating points (OPs) that prioritize either sensitivity or specificity are shown.

Although the algorithm predicts the ordinal grade of a heart murmur, another way to view the results is to reduce the task to a binary detection of a murmur of any intensity (ie, deciding murmur vs no murmur). Figure 4 shows an ROC curve for the algorithm's performance on this binary task, compared to the prediction of the attending cardiologist as the gold standard. As a baseline, the performance of the human murmur algorithm before fine‐tuning on the dog dataset is also shown. The dog algorithm achieved an area under the ROC curve (AUC) of 0.926 (95% CI: 0.905‐0.946) and performed uniformly better compared to the baseline human algorithm across the range of probability thresholds, with a mean increase in AUC of 0.056 (95% CI: 0.036‐0.079). Two example operating points are shown: a sensitive point achieved a sensitivity of 87.9% (95% CI: 83.8%‐92.1%) and specificity of 81.7% (95% CI: 72.8%‐89.0%), whereas a specific operating point achieved a sensitivity of 80.0% (95% CI: 74.4%‐85.0%) and a specificity of 91.1% (95% CI: 0.857‐0.954).

The algorithm's sensitivity for binary detection of a murmur increased with the loudness of the murmur. The sensitivity for soft murmurs was 72.2% (95% CI: 64.1%‐81.5%), but this increased to 97.2% (95% CI: 93.7%‐100%) for moderate and 99.7% (95% CI: 97.9%‐100%) for loud murmurs.

Table 4 further shows how the algorithm's ordinal predictions relate to the gold standard expert grade, averaged over all bootstrapped runs at the sensitive operating point. The correct grade was chosen by the algorithm in 57.0% of cases (95% CI: 52.8%‐61.0%). It was common for the algorithm and expert to disagree by 1 grade. For example, 41.6% (95% CI: 25.6%‐56.2%) of cases where the expert predicted a loud murmur were predicted as a moderate murmur by the algorithm. This was particularly true for thrilling murmurs: 33.6% (95% CI: 17.6%‐50.8%) were predicted as thrilling by the algorithm, whereas 47.3% (95% CI: 30.4%‐65.2%) were predicted as loud. However, large (>1 grade) errors were uncommon. The grade predicted by the algorithm was within 1 grade of the cardiologist's label in 95.2% of cases (95% CI: 93.3%‐96.9%).

**TABLE 4:** Breakdown of algorithm murmur intensity predictions by target expert murmur intensity grade.

Algorithm prediction	Proportion of algorithm predictions of a given murmur intensity within each expert‐annotated murmur grade				
	None	Soft	Moderate	Loud	Thrilling
None	0.817	0.278	0.028	0.003	0.005
Soft	0.166	0.490	0.262	0.099	0.030
Moderate	0.014	0.202	0.487	0.416	0.155
Loud	0.002	0.030	0.210	0.413	0.473
Thrilling	0.000	0.000	0.013	0.068	0.336

*Note*: Each column represents a full proportion of recordings with the associated expert grade. For example, the first cell shows that 81.7% of recordings labeled as normal by the expert are also labeled as normal by the algorithm.

Table 5 shows the sensitivity of the murmur prediction at picking up the most common diseases in the dataset at the sensitive operating point. A murmur was predicted for 91.4% (95% CI: 85.6%‐96.3%) of MMVD patients. The sensitivity was similarly high for pulmonic stenosis (98.5%; 95% CI: 89.3%‐100%), aortic stenosis (99.5%; 95% CI: 91.7%‐100%) and patent ductus arteriosus (87.7%; 95% CI: 62.5%‐100%). 88.5% of MMVD patients were predicted to have a murmur with a location of maximal intensity including the left apex, whereas for pulmonic stenosis the modal location was the left base (88.3%). This overall distribution matches the locations annotated by the expert in Table 2.

**TABLE 5:** Diagnostic sensitivity of binary murmur detection algorithm for different structural heart diseases.

Variable	DCM	MMVD	PDA	PS	AS
Murmur algorithm sensitivity (%)	73.1 (37.5‐100.0)	91.4 (85.6‐96.3)	87.7 (62.5‐100.0)	98.5 (89.3‐100.0)	99.5 (91.7‐100.0)
Locations of maximal predicted grade					
Left apex	83.2 (50.0‐100.0)	88.5 (82.5‐93.8)	30.4 (0.0‐63.2)	18.8 (0.0‐42.9)	73.3 (40.0‐100.0)
Left base	60.2 (22.2‐100.0)	50.2 (41.5‐58.9)	88.7 (60.0‐100.0)	88.3 (69.2‐100.0)	69.8 (35.7‐95.0)
Right side	55.6 (16.7‐92.3)	47.3 (37.6‐57.7)	35.7 (7.1‐66.7)	39.2 (15.0‐66.7)	54.5 (22.2‐85.7)

*Note*: Each value is reported as mean (95% CI). A murmur for a patient was predicted if any of the patient's recording locations were predicted as having a murmur of any grade. Some patients had multiple locations with the same predicted grade, so the maximal location is shared.

Abbreviations: AS, aortic stenosis; DCM, dilated cardiomyopathy; PDA, patent ductus arteriosus; PS, pulmonic stenosis.

### 3.3 Mitral valve disease

The algorithm's predictions were further analyzed over all of the MMVD (n = 407) samples and those staged as grade A (at‐risk, n = 62). For these analyses, the murmur intensity prediction at the left apex was used because it is the expected point of maximal intensity for a mitral regurgitation caused by MMVD.

**FIGURE 5:**
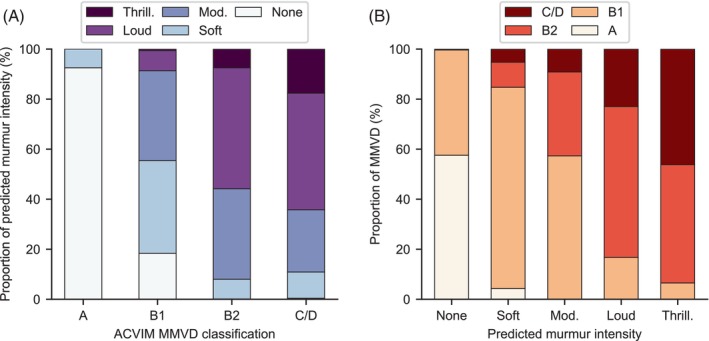
Distribution of predicted murmur intensities across patients in various MMVD stages. Each column of (A) shows the proportion of predicted murmur intensities within a specific ACVIM classification. Each column of (B) shows the proportion of ACVIM classifications within a specific murmur intensity prediction.

Figure 5 shows the breakdown of left apical murmur intensity predictions viewed from 2 perspectives. Figure 5A shows the algorithm predictions grouped by MMVD grade from gold standard echocardiography, whereas Figure 5B shows the MMVD grade grouped by the algorithm predictions. From Figure 5A, it can be seen that there was a strong positive correlation between predicted murmur intensity and the MMVD stage, as was observed previously.^4^ 9% (95% CI: 2%‐18%) of B1 cases were predicted to have a loud or thrilling murmur, which increased to 56% (95% CI: 35%‐75%) for B2 and 64% (95% CI: 33%‐91%) for congestive heart failure (C/D) cases. From Figure 5B, it can be seen that the prediction of a loud or thrilling murmur was strongly indicative of stage B2 or higher MMVD; 83% (95% CI: 68%‐96%) of loud and 94% (95% CI: 64%‐100%) of thrilling predictions had this grade on echocardiography. However, 42% (95% CI: 25%‐59%) of those predicted to have no murmur were in stage B1, but this result is affected by the low frequency of cases without a murmur (stage A) in the MMVD dataset.

**FIGURE 6:**
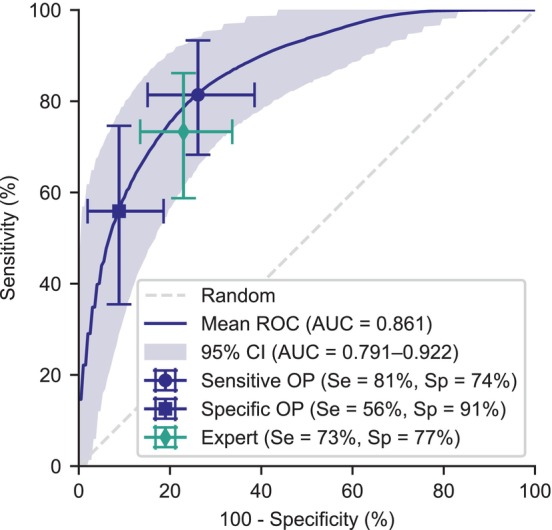
ROC curve for differentiation of B1 and B2 MMVD on the preclinical sample, where the continuous test variable is the algorithm's predicted probability of a loud or thrilling murmur. Mean and 95% confidence intervals (generated through repeated runs and iterative bootstrapping) are shown. Two example operating points (OPs) prioritize sensitivity or specificity. An operating point for the expert is shown, if all patients with a loud or thrilling left apical murmur were staged as B2.

As shown in Figure 1, the full dataset was reduced into a preclinical MMVD sample to reflect the realistic clinical situation where an asymptomatic dog with a heart murmur (stage B) needs to be further classified into B1 or B2. This is a binary classification problem, with echocardiographic criteria as the gold standard, and the results of the left apex murmur intensity prediction on this task are shown on the ROC curves in Figure 6 for the full preclinical MMVD sample (n = 343). The neural network outputs a probability distribution over the murmur grade. To generate a continuous test variable for ROC analysis, we calculate the probability that the murmur is ‘loud or greater’ by adding together the predicted probabilities from the neural network that the murmur is loud and thrilling. The model achieves an average ROC of 0.861 (95% CI: 0.791‐0.922).

If a predicted loud or thrilling murmur was used to decide stage B2, the specific operating point shown in Figure 6 was achieved. However, the continuous probability output from the neural network allows the model to operate at lower thresholds to improve sensitivity. An example sensitive operating point is shown in Figure 6 that achieves a sensitivity of 81.4% (95% CI: 68.3%‐93.3%) and a specificity of 73.9% (95% CI: 61.5%‐84.9%). On the same dataset, the expert predicting a murmur intensity of a loud or greater murmur achieves a sensitivity of 73.3% (95% CI: 58.7%‐86.2%) and a specificity of 77.0% (95% CI: 66.4%‐86.5%). At the sensitive operating point, the algorithm improves upon the expert murmur intensity prediction by an average sensitivity of 8.1%, but the 95% CIs for this difference include the null hypothesis of zero change (95% CI: −7.7%‐24.6%).

**FIGURE 7:**
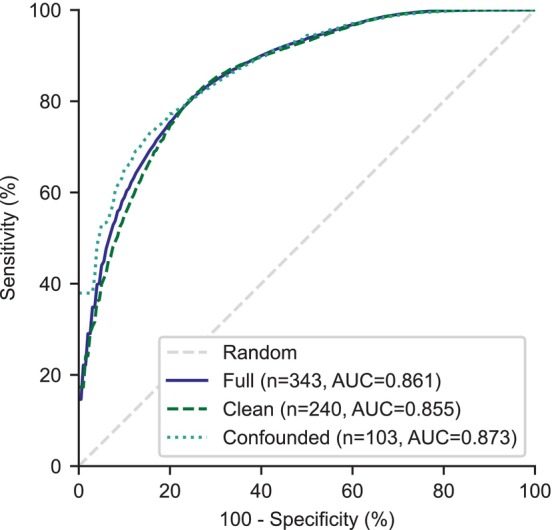
ROC curve for differentiation of B1 and B2, where the continuous test variable is the algorithm's predicted probability of a loud or thrilling murmur, shown across the 3 samples of the preclinical MMVD dataset.

The results were further analyzed by splitting the preclinical dataset into clean and confounded samples to investigate if the effect of extremes of age, weight, or pimobendan administration affected the performance of the algorithm. However, Figure 7 shows that the algorithm's performance was consistent across the clean (AUC, 0.855; 95% CI: 0.767‐0.927), confounded (AUC, 0.873; 95% CI: 0.729‐0.970), and combined full preclinical MMVD sample (as reported above; AUC, 0.861; 95% CI: 0.791‐0.922).

## 4 DISCUSSION

We used a novel dataset to show that a machine‐learning algorithm can detect and grade heart murmurs in dogs with high accuracy compared with an expert. Furthermore, these murmur predictions are sensitive to common cardiac diseases and useful in differentiating stage B1 and B2 preclinical MMVD. These predictions could help select dogs for timely referral for echocardiography and pimobendan treatment while minimizing unnecessary and costly referrals for mildly affected dogs. This study is at the development and validation stage of artificial intelligence (AI) in medical research.^34^

To the best of our knowledge, our study presents the largest database of annotated heart sound recordings from dogs collected to date. This large dataset allowed robust training of a machine‐learning algorithm that could accurately detect murmurs at a range of intensities, with particularly high sensitivity for moderate or louder cases and congenital heart diseases such as pulmonic stenosis. Differentiation between loud and thrilling murmurs by sound alone was expected to be challenging, because, by definition, thrilling murmurs are detected by chest palpation, and may not exhibit a louder sound intensity in the recordings analyzed by the algorithm.

A transfer learning approach was used to adapt a murmur‐detection algorithm for use in humans to dog heart sound data. The algorithm used in humans generalized well to murmur predictions on another species, but fine‐tuning on dog data significantly improved performance. This result was expected because, physiologically, human and dog hearts are similar. Therefore, a transfer‐learning approach could be used to port an algorithm from humans to dogs, or vice versa, for detecting abnormal heart sounds in other critical diseases such as cardiomyopathy. This approach is particularly useful where only small training sets are available.

A potential advantage of deploying an automated murmur detection algorithm is minimizing inter and intraobserver variability in heart murmur grading. However, it was not possible to investigate this effect in our study because 1 cardiologist reviewed each case, instead of a board of reviewers. Fluctuations in grading scales among reviewers may cause the current results to represent a pessimistic view of the algorithm's performance, because it was common for the algorithm and single expert to disagree by 1 grade.

We confirmed the results of previous work that murmur intensity at the left apex, graded by either the expert or the algorithm, has predictive value in differentiating B1 and B2 MMVD. A previous study reported that 30% of B1 dogs have a loud or thrilling murmur, which increases to 70% in B2 cases.^2^ Similarly, another study reported that 25% of stage B1 and 91% of stage B2 dogs have a loud or thrilling murmur.^3^ Both studies investigated combining point‐of‐care clinical measurements in multivariable models to differentiate B1 and B2, with N‐terminal pro‐B‐type natriuretic peptide (NT‐proBNP) found to have the best discriminatory ability. Compared with these studies, the algorithm we used is promising because it only used sound recordings as input variables yet achieved an AUC of 0.861, which is similar to the AUC of 0.84 achieved previously.^2^ However, the algorithms were evaluated on different datasets, and thus a direct comparison cannot be made. Our study did not collect biomarker information, and so a direct comparison between NT‐proBNP and murmur algorithm predictions could not be performed using our dataset. Both previous studies also noted that although murmur intensity is a key variable, its subjectivity among observers is a limiting factor for future deployment. The algorithm presented here can automate this murmur grading input and enable reliable deployment of these multivariable algorithms in the hands of non‐experts. The efficacy of the algorithm could be tested in a prospective study that collects both biomarker information and electronic stethoscope recordings to directly compare variables and develop a new model trained on heart sound and biomarker information.

The most important limitation of these results is that the preclinical MMVD sample contained only 217 B1 and 126 B2 cases. The substantial interpatient variability in cases led to a wide CI in the preclinical MMVD results, particularly for sensitivity. Further data collection, particularly of clean B2 cases, is needed to provide more statistical certainty.

To progress toward practical deployment, further data collection also should be focused on prospectively evaluating the algorithm's performance in the intended clinical environment of first opinion practice. Patients in our current work were recruited in specialist referral centers to enable the collection of a diverse, high‐quality dataset that included gold standard echocardiography. However, doing so meant that the proportion of dogs with severe disease receiving pimobendan was higher than in first opinion practice. Nevertheless, the performance on the clean sample, likely most representative of first opinion practice, was similar to the full preclinical sample. The data also was collected by cardiologist experts with substantial auscultation experience, but for maximum impact, the algorithm would be deployed in first opinion practices and used by general veterinarians and nurses. In these cases, worse placement of the stethoscope and higher environmental noise may lead to decreased signal quality recordings that may impact performance and cause discrepancies with predictions of a human expert. Algorithms to assess the signal quality of heart sound recordings from humans previously have been discussed^35^ and could be similarly fine‐tuned for dogs. Our study did not indicate the auscultation performance of first‐opinion practice veterinarians. Future work could directly compare the algorithm's performance and non‐expert human assessment. This approach could operate as a reader study, where positive predictions from the algorithm and a randomly selected set of controls are referred for auscultation and echocardiographic assessment by a board of cardiologists, enabling a comparison of general veterinarian and algorithm performance in a realistic setting.

Further evaluation also should investigate the correct probability threshold for a referral for echocardiography and possible pimobendan treatment. In our study, both a sensitive and a specific operating point were presented alongside threshold‐independent metrics such as the ROC curve. In first opinion practice, a sensitive operating point may be desirable to limit false negative cases and subsequent delayed treatment. However, false positives still result in unnecessary anxiety and a waste of both time and money for the owners. This trade‐off may need to be adjusted for individual practices, especially in areas of low income where access to echocardiography may be limited.

In conclusion, we showed that a proof‐of‐concept machine‐learning algorithm can be trained to use electronic stethoscope recordings to accurately detect heart murmurs in dogs and assist in the staging of preclinical MMVD. The algorithm has the potential to lower the skill barrier of auscultation and improve the early detection and management of common cardiac diseases. Future work will focus on prospective evaluation in primary care clinical environments where the algorithm may have the most important impact.

## CONFLICT OF INTEREST DECLARATION

Jose Novo Matos serves as Associate Editor for the Journal of Veterinary Internal Medicine. He was not involved in the review of this manuscript. Andrew McDonald and Anurag Agarwal are inventors on a patent application relating to elements of the algorithms in this work. No other authors declare a conflict of interest.

## OFF‐LABEL ANTIMICROBIAL DECLARATION

Authors declare no off‐label use of antimicrobials.

## INSTITUTIONAL ANIMAL CARE AND USE COMMITTEE (IACUC) OR OTHER APPROVAL DECLARATION

Approved by the Ethics and Welfare Committee of the Department of Veterinary Medicine, University of Cambridge (CR582, CR372).

## HUMAN ETHICS APPROVAL DECLARATION

Authors declare human ethics approval was not needed for this study.

## Supporting information


**Table S1:** Summary of how this study adheres to the STARD 2015 guidelines for reporting diagnostic accuracy studies.
