# Supplementary material for: A machine‐learning algorithm to grade heart murmurs and stage preclinical myxomatous mitral valve disease in dogs
Source: J Vet Intern Med. 2024 Oct 21;38(6):2994–3004. doi: 10.1111/jvim.17224 (PMC11586535; doi:10.1111/jvim.17224)
Supplement: Supplementary file 1 — Table S1: Summary of how this study adheres to the STARD 2015 guidelines for reporting diagnostic accuracy studies. [file JVIM-38-2994-s001.docx]

**Supporting Table 1.** Summary of how this study adheres to the STARD 2015 guidelines for reporting diagnostic accuracy studies.

| **No.** | **STARD Item** | **Study approach** |
| --- | --- | --- |
| 1 | Identification as a study of diagnostic accuracy using at least one measure of accuracy (such as sensitivity, specificity, predictive values or AUC) | Measures of diagnostic accuracy are included in the abstract. |
| 2 | Structured summary of study design, methods, results and conclusions (for specific guidance, see STARD for Abstracts) | A structured summary is included in the abstract. |
| 3 | Scientific and clinical background, including the intended use and clinical role of the index test | See Section 1 (Introduction). Detection and grading for heart murmurs to aid in staging of preclinical mitral valve disease. |
| 4 | Study objectives and hypotheses | See Section 1 (Introduction). |
| 5 | Whether data collection was planned before the index test and reference standard were performed (prospective study) or after (retrospective study) | Retrospective study. Data collection was performed before the design of the AI-enabled index test. |
| 6 | Eligibility criteria | Allcomers attending referral centres for routine echocardiography. |
| 7 | On what basis potentially eligible participants were identified (such as symptoms, results from previous tests, inclusion in registry) | Participants prospectively recruited from those attending routine care. |
| 8 | Where and when potentially eligible participants were identified (setting, location and dates) | See Section 2.1. |
| 9 | Whether participants formed a consecutive, random, or convenience series | Convenience sample, participants screened by cardiologists at point of exam |
| 10a | Index test, in sufficient detail to allow replication | The index test (the machine learning algorithm) is described in Section 2.4 (Machine learning analysis). |
| 10b | Reference standard | The two reference standards (cardiologist murmur grading) and echocardiography are described in Section 2.2 (Clinical evaluation). |
| 11 | Rationale for choosing the reference standard (if alternatives exist) | Echocardiography is an accepted gold-standard for cardiac evaluation: radiography not performed routinely. See Section 2.2. |
| 12a | Definition of and rationale for test positivity cut-offs or result categories of the index test, distinguishing prespecified from exploratory | Threshold-independent results have been shown as receiver operating characteristic curves. All cutoffs are exploratory. |
| 12b | Definition of and rationale for test positivity cut-offs or result categories of the reference standard, distinguishing prespecified from exploratory | Categories for murmur grading based on established literature whereas MMVD grading is based on ACVIM consensus guidelines. See Section 2.2. |
| 13a | Whether clinical information and reference standard results were available to the performers or readers of the index test | The index test was performed by a machine learning algorithm which had no knowledge of the reference standard. |
| 13b | Whether clinical information and index test results were available to the assessors of the reference standard | Index test results were not available to the performers of the reference standard. Routine clinical information was available.1 |
| 14 | Methods for estimating or comparing measures of diagnostic accuracy | Please see measures detailed in Statistical Analysis. |
| 15 | How indeterminate index test or reference standard results were handled | No indeterminate index tests or reference standards were used |
| 16 | How missing data on the index test and reference standard were handled | See Section 3.1 (Dataset). All dogs received the key left apex stethoscope recording and echocardiographic assessment. |
| 17 | Any analyses of variability in diagnostic accuracy, distinguishing prespecified from exploratory | Please see section 3.2, 3.3, and Tables 4 and 5, which breakdown accuracy by key diseases and severities. All analysis was retrospective and exploratory. |
| 18 | Intended sample size and how it was determined | See Section 3.1 (Dataset). Sample size was exploratory. |
| 19 | Flow of participants, using a diagram | See Figure 1. |
| 20 | Baseline demographics and clinical characteristics of participants | See Table 3.2. |
| 21a | Distribution of severity of disease in those with the target condition | See Table 3 for the breakdown of MMVD dogs into stages A, B1, B2, and C/D. |
| 21b | Distribution of alternative diagnoses in those without the target condition | See Table 2 for alternative cardiac disease present, and their relative murmurs. |
| 22 | Time interval and any clinical interventions between index test and reference standard | Stethoscope and echocardiographic examination was performed on the same day. |
| 23 | Cross tabulation of the index test results (or their distribution) by the results of the reference standard | See Tables 4 and 5, and Figure 5, for a breakdown of the predictions compared to the reference. |
| 24 | Estimates of diagnostic accuracy and their precision (such as 95% CIs) | See Figures 4, 6 and 7 for diagnostic accuracy with 95% CIs. |
| 25 | Any adverse events from performing the index test or the reference standard | No adverse events from either echocardiography or stethoscope examination. |
| 26 | Study limitations, including sources of potential bias, statistical uncertainty and generalisability | Limitations (including limited sample size, observer variability) are presented in Section 4 (discussion). |
| 27 | Implications for practice, including the intended use and clinical role of the index test | Potential clinical use of the test is discussed in Section 4 (discussion) |
| 28 | Registration number and name of registry | See Section 2.1. for local registration numbers of the study. |
| 29 | Where the full study protocol can be accessed | Section 2.2 gives a detailed protocol. Formal ethical protocol will be made available by the authors on reasonable request. |
| 30 | Sources of funding and other support; role of funders | Detailed in manuscript disclosure. |
